# Metabolite-disease interaction prediction based on logistic matrix factorization and local neighborhood constraints

**DOI:** 10.3389/fpsyt.2023.1149947

**Published:** 2023-06-05

**Authors:** Yongbiao Zhao, Yuanyuan Ma, Qilin Zhang

**Affiliations:** ^1^National Engineering Research Center for E-Learning, Central China Normal University, Wuhan, Hubei, China; ^2^School of Computer Engineering, Hubei University of Arts and Science, Xiangyang, Hubei, China

**Keywords:** logistic matrix factorization, neighborhood regularization, metabolite-disease interaction, association prediction, vicus matrix

## Abstract

**Background:**

Increasing evidence indicates that metabolites are closely related to human diseases. Identifying disease-related metabolites is especially important for the diagnosis and treatment of disease. Previous works have mainly focused on the global topological information of metabolite and disease similarity networks. However, the local tiny structure of metabolites and diseases may have been ignored, leading to insufficiency and inaccuracy in the latent metabolite-disease interaction mining.

**Methods:**

To solve the aforementioned problem, we propose a novel metabolite-disease interaction prediction method with logical matrix factorization and local nearest neighbor constraints (LMFLNC). First, the algorithm constructs metabolite-metabolite and disease-disease similarity networks by integrating multi-source heterogeneous microbiome data. Then, the local spectral matrices based on these two networks are established and used as the input of the model, together with the known metabolite-disease interaction network. Finally, the probability of metabolite-disease interaction is calculated according to the learned latent representations of metabolites and diseases.

**Results:**

Extensive experiments on the metabolite-disease interaction data were conducted. The results show that the proposed LMFLNC method outperformed the second-best algorithm by 5.28 and 5.61% in the AUPR and F1, respectively. The LMFLNC method also exhibited several potential metabolite-disease interactions, such as “Cortisol” (HMDB0000063), relating to “21-Hydroxylase deficiency,” and “3-Hydroxybutyric acid” (HMDB0000011) and “Acetoacetic acid” (HMDB0000060), both relating to “3-Hydroxy-3-methylglutaryl-CoA lyase deficiency.”

**Conclusion:**

The proposed LMFLNC method can well preserve the geometrical structure of original data and can thus effectively predict the underlying associations between metabolites and diseases. The experimental results show its effectiveness in metabolite-disease interaction prediction.

## 1. Introduction

Metabolites, the final product of the cell regulation process, are also regarded as the final response of a biological system to genetic or environmental changes (1, 2). Changes in metabolite levels are important markers of disease development, directly reflecting the physiological state of the human body and metabolic abnormalities. Nicholson et al. (3) pointed out that the level of metabolites reflects the effect of the human body on drug treatment and can be used as an important indicator of susceptibility and disease rehabilitation. Disease-related metabolite identification can improve clinical diagnosis and deepen the understanding of pathological mechanisms. Therefore, it is a critical task and challenge in precision medicine and biology (4).

Researchers have developed numerous methods, mostly experimental or computational, to mine the relationship between metabolites and diseases. For example, Ouyang et al. (5) discovered that metabolites (e.g., isoleucine, triglyceride, leucine, and creatinine) revealed significantly higher in the serum of pancreatic cancer patients than those in the serum of healthy controls by using 1H NMR spectroscopy and principal component analysis. Reinke et al. (6) did a metabolomics analysis to identify different metabotypes of asthma severity and found that 15 out of 66 identified serum metabolites were significantly changed with asthma. Ibanez et al. (7) developed a non-targeted metabolomics method to detect differences in metabolites in cerebrospinal fluid samples from subjects with different cognitive states associated with the progression of Alzheimer’s disease. Further, Wang et al. (8) proposed a metabolomics method based on ultra-high performance liquid chromatography–mass spectrometry to identify 13 potential biomarkers, such as succinic acid (Canavaninosuccinate) and glycochenodeoxycholic acid, which effectively distinguished patients with hepatocellular carcinoma or cirrhosis from the control group and provided important indicators for the early diagnosis and screening of patients with liver cancer. Compared with traditional experimental methods, computational approaches are relatively convenient and economical and are now more important in the field of disease-metabolite interaction relationship prediction.

Recently, some researchers have used machine learning methods to predict the interactions between metabolites and diseases (1, 2, 9–12). The majority of these methods work as follows: First, a metabolite-related heterogeneous network is built by integrating multi-omics information; second, the candidate metabolites are scored via a random walk-based method (4, 9, 13); finally, the ranking of disease-related metabolites is obtained according to the score. These methods comprehensively consider the information from multiple sources, including the genome, phenotype, and metabolic pathway, but they ignore the noise and outliers in the metabolite interaction network, undermining the reliability of the final prediction. An effective solution is to utilize the neighbor information of disease (metabolite) nodes. It benefits in two aspects: (i) effectively reducing the computational complexity, especially the construction of large-scale node similarity networks, and (ii) largely eliminating noise and interference information.

Several studies have verified that compared with the global similarity network, the local structure information (neighbors) of nodes can significantly improve the algorithm’s performance. Ma et al. (12) adopted the nearest neighbor regularization to eliminate the noise information in the metabolite-disease interaction network, and obtained good prediction results, which proved the effectiveness of the local structure information in the prediction of metabolite-disease interaction. Zhou et al. (14) achieved the accurate classification of unlabeled nodes by introducing local neighbor information. The construction strategy of the nearest neighbor graph determines the algorithm’s performance. The nearest neighbor constraint usually adopts Laplacian graph regularization. However, Wang et al. (15) designed the local spectral matrix, called Vicus, which can outperform the Laplacian matrix in some scenarios.

In addition, LMF (logical matrix factorization) has been successfully applied in the biological interaction prediction. Johnson (16) demonstrated the advantages of logical matrix factorization in modeling unobserved connections, which was realized by setting different weights for positive and negative samples. Liu et al. (17) predicted the drug-target interaction by combining the neighbor structure of nodes and the logical matrix factorization algorithm.

In this paper, we propose a novel algorithm based on logical matrix factorization and considering the local structure information (using the aforementioned spectral matrix) to predict metabolite-disease interactions. The paper’s main contributions are as follows.

- Integrating multisource information, such as disease description information from medical subject headings (MeSH) and disease-gene interaction information to build a disease similarity network. Multi-source information fusion can avoid the unreliability and inaccuracy in results caused by measurement errors and noises from a single data source, and it can describe the correlation between nodes more comprehensively;
- The impact of noise and outliers is largely eliminated by employing the logical matrix factorization and local neighbor structure information. The neighbor’s matrix constructed by the label diffusion algorithm has obvious advantages over the traditional Laplacian matrix. The experimental results show that the proposed method was superior to the baseline and state-of-the-art algorithms on the metabolite-disease dataset. The performance was improved by 5.28 and 5.61% in AUPR and F1, respectively;
- The proposed method is easily extended to other biological problems, such as phage-host interaction prediction and metabolite-drug interaction prediction.

## 2. Materials and methods

### 2.1. Dataset

The collected data fall into three categories:

- Disease-related data, which were downloaded from the Comparative Toxicogenomic Database (CTD) (18). Data sources include: ① the human disease medical dictionary, which consists of 12,988 disease names, MeSH ID, Online Mendelian Inheritance in Man (OMIM) ID, disease synonyms, and the tree-structured disease representation; ② 25,114,553 interactions between 46,045 genes and 7,163 diseases; ③ 1,727,119 interactions between 13,126 Gene Ontology Biological Processes (GO BPs) and 7,116 diseases;
- Metabolite-related data, which were collected from the Human Metabolome Database (HMDB) (19). The data include 814,427 interactions between 5,643 genes and 24,444 metabolites. Furthermore, the functional similarity network of metabolites was derived from the human gene interaction network (1);
- Metabolite-disease interaction data, which were also obtained from the HMDB (19). Originally, the data contained 24,722 interactions between 649 diseases and 22,265 metabolites. By removing diseases without OMIM ID and semantic similarity and metabolites lacking functional similarity, we shrank that figure to 3,360 interactions between 337 diseases and 1,444 metabolites.

### 2.2. Problem formalization

In this article, the set of metabolites is denoted by $M={\left\{m_{i}\right\}}_{i=1}^{n}$ , and the set of diseases is denoted by $D={\left\{d_{j}\right\}}_{j=1}^{m}$ , where *n* and *m* are the number of metabolites and diseases, respectively. The known metabolite-disease interactions are represented as an n×m binary matrix ( $Y\in R^{n\times m}$ ), where $y_{ij}=1$ if a metabolite ( $m_{i}$ ) has been observed to interact with a disease ( $d_{j}$ ); otherwise, $y_{ij}=0$ .This study aimed to solve the problem of predicting the interaction probability of a disease-metabolite pair, and it subsequently ranked the candidate disease-metabolite pairs based on these probabilities in descending order. Thus, the top-ranked pairs can be viewed as latent interactions.

### 2.3. Metabolite-disease interaction prediction process based on logical matrix factorization and local neighborhood constraints

**Figure 1:**
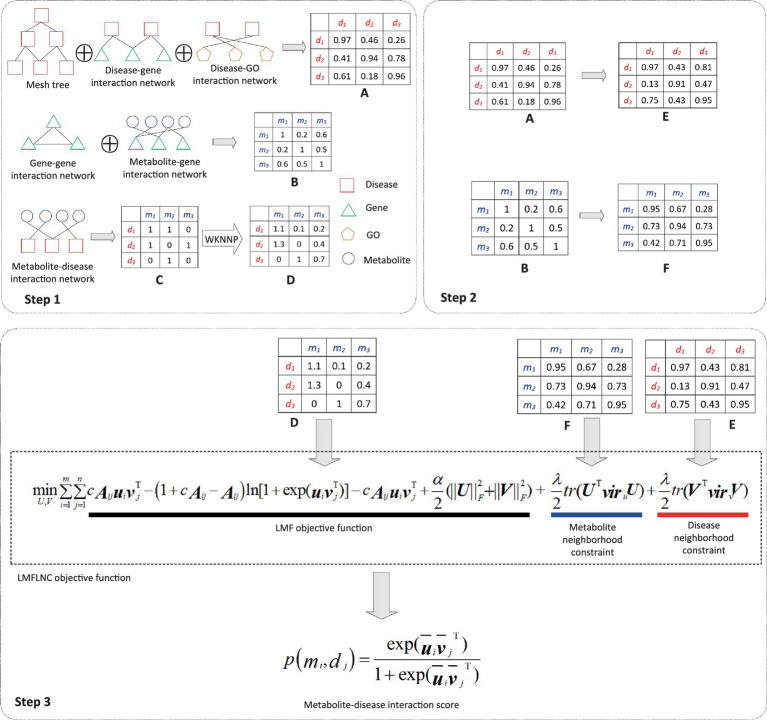
Flowchart of metabolite-disease interaction prediction with the LMFLNC. In step1, the disease-disease similarity matrix *A* is constructed by integrating the disease-related data disease-gene interactions, disease-GO interactions, and the MeSH tree with clusDCA. Similarly, the metabolite-metabolite similarity matrix *B* is built from gene–gene associations, metabolite-gene interactions; Running WKNNP on the metabolite-disease interaction matrix *C* generates the completed metabolite-disease interaction matrix *D*; In step 2, the local spectral matrices of diseases and metabolites *E* and *F* are obtained based on the disease-disease similarity matrix and metabolite-metabolite matrix, respectively. Finally, in step 3, the proposed LMFLNC was used to predict the metabolite-disease interaction scores.

The prediction process, as demonstrated in Figure 1, can be divided into three subprocesses:

- The disease-disease similarity network is constructed by integrating the disease-related data (disease-gene interactions, disease-GO interactions, and the MeSH tree). Similarly, the metabolite-metabolite similarity network is built from the metabolite-related data (gene–gene associations, metabolite-gene interactions. Due to its highly sparse and noisy, the metabolite-disease interaction data is smoothed via WKNNP (20).
- The local spectral matrices of diseases and metabolites are computed based on the disease-disease similarity network and metabolite-metabolite network, respectively.
- The metabolite-disease interaction probabilities are computed by feeding the modified metabolite-disease interaction matrix, metabolite local spectral matrix, and disease local spectral matrix into the proposed logical matrix factorization model based on the local nearest neighbor constraint (LMFLNC).

Two crucial steps in the prediction process need further explanations.

(i) Disease-disease similarity network construction.

To obtain the comprehensive and accurate similarity between diseases, multiple data source of diseases including disease MeSH descriptors, disease–GO biological process interaction networks and disease–gene interaction networks are integrated. We employs the MultiSourcDSim model presented in (21) to calculate the semantic similarity of diseases. Specifically, for MeSH descriptors (22), we firstly construct a directed acyclic graph (DAG) to describe the relationships between any two diseases. Secondly, the probability of a disease term is calculated with its frequency occurring in the association dataset (Eqs. 1–2). Finally, the disease similarity (Eq. 3) is calculated with Lin’s method (23).


(1) $$f\left(t\right)=\mathrm{self}\left(t\right)+\underset{tc\in \mathrm{children}\left(t\right)}{\sum }f\left(tc\right)$$

(2) $$\mathrm{prob}\left(t\right)=\frac{f\left(t\right)}{N}\text{.}$$

(3) $$\mathrm{score}\left(t_{1},t_{2}\right)=\max_{t\in LCA\left(t_{1},t_{2}\right)}\left(\frac{2\times \log\mathrm{prob}\left(t\right)}{\log\mathrm{prob}\left(t_{1}\right)+\log\mathrm{prob}\left(t_{2}\right)}\right)$$

where $\mathrm{self}\left(t\right)$ is the number of disease term *t*, *tc* is a direct child of *t*. $f\left(t\right)$ is the frequency at which *t* occurs in the single association dataset. *N* is the frequency of the root node term. $LCA\left(t_{1},t_{2}\right)$ is the set of least common ancestors of term $t_{1}$ and $t_{2}$ . $\mathrm{score}\left(t_{1},t_{2}\right)$ denotes the semantic similarity score between disease terms $t_{1}$ and $t_{2}$ . For the other two data source, the similarity score is used to compute the disease similarity network.

(ii) Metabolite-metabolite similarity network construction.

With metabolite-gene interaction data, the similarity between any two genes, $g_{i}$ and $g_{j}$ , can be measured as


(4) $$Sim\_g\left(g_{i},g_{j}\right)=|GO_{i}\cap GO_{j}|/|GO_{i}\cup GO_{j}|\text{,}$$

where *GO_i_* and *GO*_*j*_ denote the GO sets explaining $g_{i}$ and $g_{j}$ , respectively.

Similarly, the similarity between a gene ( $g_{i}$ ) and a gene set (*G*) can be defined as


(5) $$SG\left(g_{i},G\right)=\max_{g_{j}\in G}\left[Sim\_g\left(g_{i},g_{j}\right)\right]\text{.}$$

According to (24), the similarity between two metabolites, $m_{1}$ and $m_{2}$ , can be computed as


(6) $$SM\left(m_{1},m_{2}\right)=\frac{\sum_{g_{1}\in G_{1}}SG\left(g_{1},G_{2}\right)+\sum_{g_{2}\in G_{2}}SG\left(g_{2},G_{1}\right)}{\left|G_{1}|+|G_{2}\right|}\text{,}$$

where $G_{1}$ and $G_{2}$ stand for gene sets related to $m_{1}$ and $m_{2}$ , respectively; |·| denotes the set size.

The metabolite-metabolite similarity network is built via Equation (6).

### 2.4. Logical matrix factorization based on local nearest neighbor constraint

Logical matrix factorization has been successfully applied to the prediction of drug-target and virus-host interactions. In this paper, a new model based on logical matrix factorization is proposed to predict the interaction between metabolites and diseases. The main idea is to map metabolites and diseases into a shared low-dimensional latent semantic space, $r<<\min\left(n,m\right)$ . Then, the probability of interaction between metabolite $m_{i}$ and disease $d_{j}$ can be modeled by the following logical function:


(7) $$p_{ij}=\frac{\exp\left(u_{i}v_{j}^{T}\right)}{1+\exp\left(u_{i}v_{j}^{T}\right)}\text{,}$$

where $u_{i}\in R^{1\times r}$ and $v_{j}\in R^{1\times r}$ are latent representations of metabolite $m_{i}$ and disease $d_{j}$ , respectively.

In logical matrix factorization, the known or experimentally verified interactions are usually more informative, so they are usually assigned higher weights than those unknown ones. Each metabolite-disease interaction is regarded as $c\left(c\geq 1\right)$ positive sample, and each unknown metabolite-disease pair is regarded as a single negative sample. c is used to control the importance level of the observed interactions, which was empirically set to 2 in the subsequent experiments.

Assuming that each training sample is independent, according to the maximum likelihood estimation, the following probability representation can be obtained:


(8) $$\begin{array}{l} p\left(A|U,V\right)=\underset{1\leq i\leq m,1\leq j\leq n,A_{ij}=1}{\prod }{\left[p_{ij}^{A_{ij}}{\left(1-p_{ij}\right)}^{1-A_{ij}}\right]}^{c}\\ \;\times \underset{1\leq i\leq m,1\leq j\leq n,A_{ij}=0}{\prod }p_{ij}^{A_{ij}}{\left(1-p_{ij}\right)}^{1-A_{ij}} \end{array}\text{.}$$

where ***A*** represents the known metabolite-disease interaction matrix; ***U*** and ***V*** represent the decomposed the metabolite and disease latent semantic matrices, respectively; *m* is the number of metabolites; *n* is the number of diseases. The logarithm of $p\left(A|,U|,V\right)$ can be inferred by combining Equation (7) with Equation (8):


(9) $$\begin{array}{l} \log\left[p\left(A|U,V\right)\right]=\overset{m}{\underset{i=1}{\sum }}\overset{n}{\underset{j=1}{\sum }}cA_{ij}u_{i}v_{j}^{T}-\left(1+cA_{ij}-A_{ij}\right)\\ \;\ln\left[1+\exp\left(u_{i}v_{j}^{T}\right)\right]\text{.} \end{array}$$

Equation (9) is also called the basic LMF objective function. The latent representation matrices ***U*** and ***V*** can be estimated by maximizing this function.

To improve the performance of the logical matrix factorization algorithm, researchers (12, 17) introduced the local neighbor constraint. They sorted the nodes by their similarities to find neighbor nodes, but they ignored the diffusion and propagation of label information carried by neighbor nodes, which limited the performance enhancement. In this study, inspired by the idea of a local spectral matrix, the Vicus matrix (15), we obtained the following objective function by using the Vicus matrix to constrain Equation (9):


(10) $$\begin{array}{l} \log\left[p\left(A|U,V\right)\right]=\overset{m}{\underset{i=1}{\sum }}\overset{n}{\underset{j=1}{\sum }}cA_{ij}u_{i}v_{j}^{T}-\left(1+cA_{ij}-A_{ij}\right)\;\ln\left[1+\exp\left(u_{i}v_{j}^{T}\right)\right]\\ \;+\lambda /2\left[tr\left(U^{T}vir_{u}U\right)+tr\left(V^{T}vir_{v}V\right)\right] \end{array}\text{,}$$

where λ is the regularization parameter to balance between the factorization error and the local spatial structure preservation; $vir_{u}$ and $vir_{v}$ represent the local spectral matrices of metabolites and diseases, respectively, whose calculation process is as follows:

Let $X=\left\{x_{1},x_{2},\cdots ,x_{n}\right\}$ be the set of data points, W be the weighted network constructed from X with X as the vertex set and the similarities among X as the weight set; $x_{i}$ be the *i*th data point in X ; the *i*th vertex in W , $N\left(i\right)$ be the neighbors of $x_{i}$ , whose size is K ; and *C* be the number of clusters.

First, for node $x_{i}$ , subnet $W_{i}\left(ver_{i},\varepsilon_{i}\right)$ is extracted from W , where the vertex set $ver_{i}=N\left(i\right)\cup x_{i}$ , and $\varepsilon_{i}$ is the edge set. Through the label diffusion algorithm (14), the label indicator vector is reconstructed as


(11) $$F_{ver_{i}}^{k}=\left(1-\alpha \right){\left(I-\alpha S_{i}\right)}^{-1}q_{ver_{i}}^{k},1\leq k\leq C\text{,}$$

where α is a constant between 0 and 1, which is set to 0.9, as suggested in (24); $q_{ver_{i}}^{k}$ is the clustering indicator vector reflecting the scaling of subnet $W_{i}$ ; $S_{i}$ represents the standardized transition matrix of $W_{i}$ , defined as $S_{i}\left(u,t\right)=W_{i}\left(u,t\right)/\overset{K+1}{\underset{l=1}{\sum }}W_{i}\left(u,l\right)$ .

Second, $q_{ver_{i}}^{k}$ is estimated by $F_{ver_{i}}^{k}$. Let ${\bar{q}}_{ver_{i}}^{k}=F_{ver_{i}}^{k}\left[K+1\right]$ indicate the likelihood that data point *i* belongs to cluster *k*. The next task is to maximize the concordance between ${\bar{q}}_{ver_{i}}^{k}$ and $q_{ver_{i}}^{k}$ . Let ${\bar{q}}_{ver_{i}}^{k}=\beta_{i}q_{ver_{i}}^{k}$ , where $\beta_{i}\in R^{K+1}$ is the row of matrix $\left(1-\alpha \right){\left(I-\alpha S_{i}\right)}^{-1}$ , which represents the convergence state of label diffusion. Thus, ${\bar{q}}_{ver_{i}}^{k}$ can be estimated as


(12) $${\bar{q}}_{ver_{i}}^{k}\approx \frac{\beta_{i}\left[1:K\right]q_{N\left(i\right)}^{k}}{1-\beta_{i}\left[K+1\right]}\text{,}$$

where $\beta_{i}\left[1:K\right]$ and $\beta_{i}\left[K+1\right]$ denotes the first *K* elements and the (*K + 1*)th element in $\beta_{i}$ , respectively.

Afterward, matrix ***B*** is constructed to represent the linear relationship between $q^{k}$ and ${\bar{q}}_{k}$ : ${\bar{q}}_{k}=Bq^{k}$ . It is computed as


(13) $$B_{ij}=\{\begin{array}{l} \frac{\beta_{ij}}{1-\beta_{i}\left[K+1\right]},\;x_{j}\in N\left(i\right)\\ 0,\;\;\mathrm{otherwise} \end{array}\text{.}$$

To minimize the difference between $q^{k}$ and ${\bar{q}}_{k}$ , we can define an objective function as


(14) $$\begin{array}{l} \overset{n}{\underset{i=1}{\sum }}\overset{C}{\underset{k=1}{\sum }}{\left({\bar{q}}_{i}^{k}-q_{i}^{k}\right)}^{2}=\overset{C}{\underset{k=1}{\sum }}q^{k}-{{\bar{q}}^{k}}^{2}\approx \overset{C}{\underset{k=1}{\sum }}q^{k}-B{q^{k}}^{2}\\ =\mathrm{Trace}\left[Q^{T}{\left(I-B\right)}^{T}\left(I-B\right)Q\right] \end{array}\text{.}$$

Finally, let $vir={\left(I-B\right)}^{T}\left(I-B\right)$ , which is the needed local spectral matrix. Wang et al. (15) proved that vir and the Laplacian matrix share many of the same properties. For example, they are both symmetric and positive semidefinite, with the minimum eigenvalue being 0 and the eigenvector being 1.

In logical matrix factorization, to prevent overfitting, we usually constrain the latent space matrices ***U*** and ***V*** to construct the final objective function as


(15) $$\begin{array}{c} \log\left[p\left(A|U,V\right)\right]=\overset{m}{\underset{i=1}{\sum }}\overset{n}{\underset{j=1}{\sum }}cA_{ij}u_{i}v_{j}^{T}-\left(1+cA_{ij}-A_{ij}\right)\ln\left[1+\exp\left(u_{i}v_{j}^{T}\right)\right]\\ \;+\alpha /2\left(||U||{}_{F}^{2}+||V||{}_{F}^{2}\right)+\lambda /2\left[tr\left(U^{T}vir_{u}U\right)+tr\left(V^{T}vir_{v}V\right)\right] \end{array}$$

where α denotes the regularization parameter. Here, the gradient descent algorithm is used to optimize Equation (15). Specifically, let *L* represent the objective function whose partial derivatives with respect to ***U*** and ***V*** are given as follows:


(16) $$\frac{\partial L}{\partial U}=PV+\left(c-1\right)\left(A⊙P\right)V-cAV+\left(\alpha I+\lambda vir_{u}\right)U\text{,}$$

(17) $$\frac{\partial L}{\partial V}=P^{T}U+\left(c-1\right)\left(A^{T}⊙P^{T}\right)U-cA^{T}U+\left(\alpha I+\lambda vir_{v}\right)V\text{,}$$

where ***P*** is the probability matrix defined by Equation (7), and ⊙ represents the Hadamard product of a matrix. After the latent representations of ***U*** and ***V*** have been acquired, any unknown metabolite-disease interaction probability can be predicted by Equation (7). However, in the training process, the latent vectors of some unobserved metabolites and diseases are obtained based on negative samples, which may not be accurate enough. Ma et al. (12) presented an effective solution. Let $N_{m}^{+}=\left\{m_{i}|\underset{j}{\sum }A_{ij}>0\right\}$ represent the set of metabolites interacting with any disease, and let $N_{m}^{+}\left(m_{i}\right)$ represent the set of *K* nearest neighbors of metabolites in $N_{m}^{+}$ . We set *K = 10* in this manuscript. Metabolite $m_{i}$ can be represented by a linear combination of the latent vectors of $N_{m}^{+}\left(m_{i}\right)$ , and is defined as follows:


(18) u¯i={ui,mi∈Nm+1Qmi∑k=1Kwkmuk,mi∉Nm+,

where $Q_{m_{i}}=\overset{K}{\underset{k=1}{\sum }}\alpha^{k-1}\bar{\mathrm{ConsM}}\left(m_{i},N_{i}^{k}\right)$ is a normalized term, $N_{i}^{k}\in N_{m}^{+}\left(m_{i}\right)$ denotes the *k*th neighbor of $m_{i}$ , and $\bar{\mathrm{ConsM}}$ is the binary neighbor similarity matrix. ${\bar{\mathrm{ConsM}}}_{ij}=\mathrm{Cons}M_{ij}$ if metabolite $m_{i}\in N\left(m_{j}\right)$ or metabolite $m_{j}\in N\left(m_{i}\right)$ ; otherwise, ${\bar{\mathrm{ConsM}}}_{ij}=0$ . $\alpha \in \left[0,1\right]$ is a decay factor, which is 0.9 in this paper. $w_{k}^{m}=a^{k-1}\bar{\mathrm{ConsM}}\left(m_{i},N_{i}^{k}\right)$ . Similarly, the representation of disease $d_{j}$ can be obtained:


(19) v¯j={vj,dj∈Nd+1Qdj∑k=1Kwkdvk,dj∉Nd+,

where $Q_{d_{j}}=\overset{K}{\underset{k=1}{\sum }}\alpha^{k-1}\bar{\mathrm{ConsD}}\left(d_{j},N_{j}^{k}\right)$ is a normalized term, $N_{j}^{k}$ denotes the *k*th neighbor of disease $d_{j}$ , and $w_{k}^{d}=a^{k-1}\bar{\mathrm{ConsD}}\left(d_{j},N_{j}^{k}\right)$ .

Eventually, the probability of an interaction between metabolite $m_{i}$ and disease $d_{j}$ can be rewritten as


(20) $$p\left(m_{i},d_{j}\right)=\frac{\exp\left({\bar{u}}_{i}{{\bar{v}}_{j}}^{T}\right)}{1+\exp\left({\bar{u}}_{i}{{\bar{v}}_{j}}^{T}\right)}\text{.}$$

In order to clearly demonstrate the steps of LMFLNC algorithm, we also presented its pseudocode in Table 1.

**Table 1:** The pseudocode of the LMFLNC algorithm.

Input: The metabolite-disease interaction matrix *A*; parameters *c*, *α*, *λ*
Output: The latent representation matrices, *U* and *V*
1. Calculate the disease–disease similarity matrix using Eq. (3); Calculate the metabolite–metabolite similarity matrix according to Eq. (6);
2. Calculate the spectral matrices of metabolites and diseases;
3. Calculate the modified metabolite-disease interaction matrix via WKNNP (20);
4. Initialize *U* and *V* randomly;
5. For *t = 1, ……, max_iter* do
6. Update *U* and *V* according to Adara algorithm
7. Until convergence conditions are satisfied
8. End for
9. Return *U, V*

## 3. Results and discussion

### 3.1. Experimental settings and evaluation metrics

Following the previous studies, we used the fivefold cross-validation technique for model validation in this paper. In each round, one-fifth of the known metabolite-disease interactions and all unobserved interactions (metabolite-disease pairs corresponding to elements of value 0 in the metabolite-disease interaction matrix *A*) were used for testing; the rest were used for training. AUPR, AUC, and F1 were adopted as performance evaluation. To achieve a relatively objective evaluation, we randomly ran the cross validation 20 times, over which the average values of the aforementioned metrics were taken as their final values. The model implementation and validation were realized in MATLAB R2017b (see Table 1).

### 3.2. Experimental results

To verify the superiority of the proposed LMFLNC model, we compared it with such baselines as MN-LMF (12), PROFANCY (2), WMAN (25), and MCF (13). The parameters of PROFANCY, WMAN, and MN-LMF were set to default values. For MCF, the reboot probability is set as the optimal element from $\left\{0.1,0.2,\cdots ,0.9\right\}$ . For LMFLNC, we set the number of nearest neighbors in local spectral matrices of metabolites and diseases as *K* = 15, the importance level of observed interactions *c* = 2, the neighbor regularization parameter λ=8 , and the latent space regularization parameter α=4 . The performance of the abovementioned algorithms on the metabolite-disease benchmark dataset is shown in Table 2.

**Table 2:** Performance comparison of metabolite-disease benchmark dataset.

Algorithm	AUPR	AUC	F1
WMAN	0.0151	0.6181	0.0800
PROFANCY	0.2325	0.9027	0.3066
MCF	0.0151	0.6156	0.0770
MN-LMF	0.3731	0.9659	0.4135
LMFLNC	0.3931	0.9661	0.4367

Table 2 shows that the LMFLNC algorithm outperformed the second MN-LMF algorithm in AUPR and F1 5.28 and 5.61%, respectively. Additionally, the prediction performances of WMAN and MCF methods were unsatisfactory. One possible reason is that these two methods simply focus on the known metabolite-disease interaction network and only leverage limited prior knowledge, that is, the disease similarity network. However, the LMFLNC method fully considers the similarities of metabolites and diseases at multiple levels and then adjusts the importance level of positive and negative samples (the observed metabolite-disease interaction is regarded as a positive sample. The unobserved metabolite-disease interaction is regarded as a negative sample) by parameter *c*, which improved its performance. Moreover, compared with MN-LMF, LMFLNC uses the local spectral matrices of metabolites and diseases to construct neighbor constraints, so the latent representations of metabolites and diseases generated by the logical matrix factorization were more robust. The experimental results show the potential of LMFLNC in predicting unknown metabolite-disease interactions.

### 3.3. Parameter analysis

**Figure 2:**
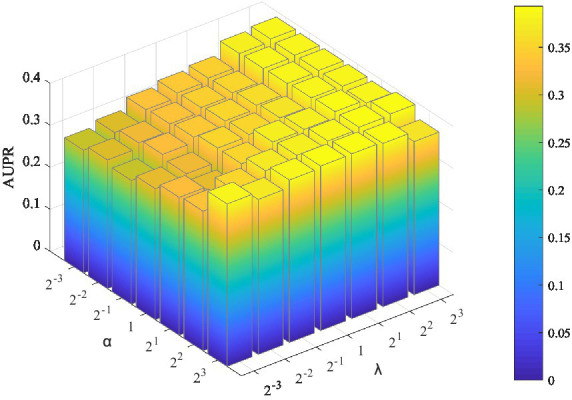
Parameter sensitivity analysis. AUPR achieves the maximum value when α =4 and λ =8.

Two parameters need to be tuned in LMFLNC: the latent space regularization parameter α and the local spectral parameter (or neighbor regularization parameter) λ ; the other ones are set by default. The grid search was employed to find the optimal parameter values. Let $\alpha \in \left\{2^{-3},2^{-2},2^{-1},2^{0},2^{1},2^{2},2^{3}\right\}$ , $\lambda \in \left\{2^{-3},2^{-2},2^{-1},2^{0},2^{1},2^{2},2^{3}\right\}$ , and the model performance over different parameter combinations was evaluated by a fivefold cross-validation. As shown in Figure 2, LMFLNC obtained the optimal prediction performance (AUPR) when α = 4 and λ = 8.

### 3.4. Case studies

We further verify the performance of LMFLNC method in this section. First, the entire dataset was used to train LMFLNC with the optimal parameters obtained above. Then, the trained LMFLNC was used to predict the interaction probabilities between all the metabolites and two example diseases, “21-Hydroxylase deficiency” and “3-Hydroxy-3-methylglutaryl-CoA lyase deficiency,” in the dataset. Table 3 displays 10 metabolites relating to the first example disease, with the probabilities listed in descending order. Similarly, Table 4 displays 15 metabolites relating to the second example disease, with the probabilities again listed in descending order.

**Table 3:** 21-Hydroxylase deficiency’ related metabolites (top 10, descend).

NO.	Metabolite ID	Metabolite name	Interaction probability	Category
1	HMDB0000374	17-Hydroxyprogesterone	0.9568	Known
2	HMDB0000053	Androstenedione	0.9308	Known
3	HMDB0000122	D-Glucose	0.9279	Known
4	HMDB0000234	Testosterone	0.9266	Known
5	HMDB0000586	Potassium	0.9241	Known
6	HMDB0000595	Hydrogen carbonate	0.9224	Known
7	HMDB0000588	Sodium	0.9056	Known
8	HMDB0000077	Dehydroepiandrosterone	0.8155	Known
9	HMDB0004030	21-Deoxycortisol	0.7693	Known
10	HMDB0000063	Cortisol	0.5896	PubMed:16439592

**Table 4:** 3-Hydroxy-3-methylglutaryl-CoA lyase deficiency-related metabolites (top 15, descend).

NO.	Metabolite ID	Metabolite name	Interaction probability	Category
1	HMDB0000122	D-Glucose	0.9776	Known
2	HMDB0000190	L-Lactic acid	0.9735	Known
3	HMDB0000051	Ammonia	0.9456	Known
4	HMDB0000754	3-Hydroxyisovaleric acid	0.9455	Known
5	HMDB0000661	Glutaric acid	0.9295	Known
6	HMDB0000062	L-Carnitine	0.9209	Known
7	HMDB0000243	Pyruvic acid	0.9152	Known
8	HMDB0000595	Hydrogen carbonate	0.8861	Known
9	HMDB0000063	Cortisol	0.8805	Known
10	HMDB0000357	3-Hydroxybutyric acid	0.8802	Known
11	HMDB0000459	3-Methylcrotonylglycine	0.8754	Known
12	HMDB0000355	3-Hydroxymethylglutaric acid	0.7789	Known
13	HMDB0000509	Senecioic acid	0.7646	Known
14	HMDB0000011	3-Hydroxybutyric acid	0.4991	PubMed:12072887
15	HMDB0000060	Acetoacetic acid	0.3614	Unconfirmed

It can be seen that all of the nine metabolites related to the disease “21-Hydroxylase deficiency” in the dataset appear in Table 3 and, more importantly, are located in the top nine. Similarly, all of the 13 metabolites related to disease “3-Hydroxy-3-methylglutaryl-CoA lyase deficiency” in the dataset are included in Table 4 and occupy the top 13. These findings demonstrate the good accuracy of LMFLNC. Note that LMFLNC also predicted that the metabolite ‘Cortisol “(HMDB0000063) were likely to interact with disease” “21-Hydroxylase deficiency” (the likelihood is 0.5896) and that metabolites “3-Hydroxybutyric acid (HMDB0000011)” and “Acetoacetic acid (HMDB0000060)” were likely to interact with disease “3-Hydroxy-3-methylglutaryl-CoA lyase deficiency” (likelihood of 0.4991 and 0.3614, respectively). Two of these three predictions have been verified, showing the potential of the LMFLNC model to discover latent metabolite-disease interactions.

In the same way, LMFLNC can compute the probabilities of diseases relating to a specific metabolite and predict new disease-metabolite interactions.

## 4. Conclusion

Existing metabolite-disease interaction prediction methods mainly leverage the global similarity network, which may be limited by noise and outliers. To solve this problem, we introduced a novel method, LMFLNC, to predict the metabolite-disease interaction. Extensive experiments were conducted on the collected dataset. The results show that the proposed LMFLNC method outperformed the baselines. LMFLNC also revealed several potential metabolite-disease interactions, such as “Cortisol (HMDB0000063),” relating to “21-Hydroxylase deficiency,” and “3-Hydroxybutyric acid (HMDB0000011)” and “Acetoacetic acid (HMDB0000060),” both relating to “3-Hydroxy-3-methylglutaryl-CoA lyase deficiency.”

Despite its promising performance, LMFLNC has the following weaknesses. (1) The predicted new metabolite-disease interactions need further verification. (2) The dataset scale, including the data quantity and type, is relatively small, and the information of metabolite structure and pathway can be incorporated to improve the performance and robustness of LMFLNC.

Our future research work will include the following: (1) exploring combining multi-kernel learning and logical matrix factorization in a study on the metabolite-disease interaction relationship and (2) exploring the application of our model in similar fields, such as microorganism-drug interactions and microorganism-metabolite interactions.

## Data availability statement

The original contributions presented in the study are included in the article/supplementary material, further inquiries can be directed to the corresponding author.

## Author contributions

YZ and YM wrote the manuscript and developed the algorithms. YM designed the concept and including the structure and content of the manuscript. YM, QZ, and YZ critically revised the manuscript. All authors reviewed and approved the final version of the manuscript.

## Funding

This work was supported by Hubei Superior and Distinctive Discipline Group of “New Energy Vehicle and Smart Transportation.”

## Conflict of interest

The authors declare that the research was conducted in the absence of any commercial or financial relationships that could be construed as a potential conflict of interest.

## Publisher’s note

All claims expressed in this article are solely those of the authors and do not necessarily represent those of their affiliated organizations, or those of the publisher, the editors and the reviewers. Any product that may be evaluated in this article, or claim that may be made by its manufacturer, is not guaranteed or endorsed by the publisher.
